# Characterization of florfenicol resistance genes in the coagulase-negative *Staphylococcus* (CoNS) isolates and genomic features of a multidrug-resistant *Staphylococcus lentus* strain H29

**DOI:** 10.1186/s13756-020-00869-5

**Published:** 2021-01-07

**Authors:** Chongyang Wu, Xueya Zhang, Jialei Liang, Qiaoling Li, Hailong Lin, Chaoqin Lin, Hongmao Liu, Danying Zhou, Wei Lu, Zhewei Sun, Xi Lin, Hailin Zhang, Kewei Li, Teng Xu, Qiyu Bao, Junwan Lu

**Affiliations:** 1 School of Laboratory Medicine and Life Science/Institute of Biomedical Informatics, Wenzhou Medical University, Chashan University Town, Wenzhou, 325035 Zhejiang China; 2 The Second Affiliated Hospital and Yuying Children’s Hospital, Wenzhou Medical University, Wenzhou, 325027 Zhejiang China; 3 Institute of Translational Medicine, Baotou Central Hospital, Baotou, 014040 China; 4 Department of Laboratory Medicine, West China Hospital, Sichuan University, Chengdu, 610041 Sichuan China

**Keywords:** Coagulase-negative staphylococci, *Staphylococcus lentus*, Florfenicol resistance genes, Whole genome, Comparative genomics analysis

## Abstract

**Background:**

With the wide use of florfenicol to prevent and treat the bacterial infection of domestic animals, the emergence of the florfenicol resistance bacteria is increasingly serious. It is very important to elucidate the molecular mechanism of the bacteria’s resistance to florfenicol.

**Methods:**

The minimum inhibitory concentration (MIC) levels were determined by the agar dilution method, and polymerase chain reaction was conducted to analyze the distribution of florfenicol resistance genes in 39 CoNS strains isolated from poultry and livestock animals and seafood. The whole genome sequence of one multidrug resistant strain, *Staphylococcus lentus* H29, was characterized, and comparative genomics analysis of the resistance gene-related sequences was also performed.

**Results:**

As a result, the isolates from the animals showed a higher resistance rate (23/28, 82.1%) and much higher MIC levels to florfenicol than those from seafood. Twenty-seven animal isolates carried 37 florfenicol resistance genes (including 26 *fexA*, 6 *cfr* and 5 *fexB* genes) with one carrying a *cfr* gene, 16 each harboring a *fexA* gene, 5 with both a *fexA* gene and a *fexB* gene and the other 5 with both a *fexA* gene and a *cfr* gene. On the other hand, all 11 isolates from seafood were sensitive to florfenicol, and only 3 carried a *fexA* gene each. The whole genome sequence of *S. lentus* H29 was composed of a chromosome and two plasmids (pH29-46, pH29-26) and harbored 11 resistance genes, including 6 genes [*cfr, fexA, ant(6)-Ia*, *aacA-aphD, mecA* and *mph(C)*] encoded on the chromosome, 4 genes [*cfr, fexA, aacA-aphD* and *tcaA*] on pH29-46 and 1 gene (*fosD*) on pH29-26. We found that the *S. lentus* H29 genome carried two identical copies of the gene arrays of *radC*-*tnpABC*-*hp*-*fexA* (5671 bp) and IS*256*-*cfr* (2690 bp), of which one copy of the two gene arrays was encoded on plasmid pH29-46, while the other was encoded on the chromosome.

**Conclusions:**

The current study revealed the wide distribution of florfenicol resistance genes (*cfr, fexA* and *fexB*) in animal bacteria, and to the best of our knowledge, this is the first report that one *S. lentus* strain carried two identical copies of florfenicol resistance-related gene arrays.

## Background

Coagulase-negative *Staphylococcus* (CoNS) are opportunistic pathogens that are found not only in animals and humans but also widely in the environment, including dust, soil, water and air. CoNS are also considered a repository of resistance genes, highlighting their threat to public health [1]. CoNS infection can lead to arthritis, cow mastitis, and even systemic infections [2]. Florfenicol is an antimicrobial widely used in veterinary medicine that acts by binding to the 50S ribosomal subunit, leading to inhibition of protein synthesis [3]. Because of its broad antibacterial activity and few adverse effects, florfenicol has been licensed exclusively for use in veterinary medicine to treat infections caused by, for example, *Pasteurella multocida*, *Staphylococcus* sp*.* and *Streptococcus* sp*.* in companion animals, farm animals and fish [4]. However, the increasing use of the antibiotics for the treatment and prevention of infectious diseases in animals has contributed to the emergence and widespread of florfenicol resistance genes among bacteria of different species or genera [5]. Reports of multidrug-resistant CoNS are also increasing, and this increased resistance of CoNS to antibiotics also limits the choice of drugs to treat infections [6]. To date, a variety of florfenicol resistance mechanisms have been characterized, including efflux pumps (*floR*, *fexA/fexB* and *pexA/pexB*) [7–11], rRNA methyltransferase (*cfr*) [12], chloramphenicol hydrolase (*estDL136*) [13], chloramphenicol acyltransferases (*catA* or *catC*) [14] and ribosomal protection proteins (*optrA* and *poxtA*) [15, 16]. In CoNS, only *cfr*, *optrA*, *poxtA* and *fexA/fexB* have been identified. The gene *cfr* was initially found on the 17.1-kb plasmid pSCFS1 from an *S. sciuri *isolate and was shown to encode an rRNA methylase mediating resistance to phenicol by methylation of the 23S rRNA. In contrast, the gene *fexA*, which encodes an efflux protein within the major facilitator superfamily (MFS), was first identified on the 34-kb plasmid pSCFS2 [17] from *S. lentus* and was shown to be part of the Tn*554*-like transposon Tn*558* [18]. *fexB*, also a phenicol exporter gene, was first identified on the pEFM-1 (35 kb in size) of *E. faecium* and pEH-1 (25.3 kb in size) of *E. hirae*, both strains with swine origins [19]. The genes *optrA* and *poxtA* encode ribosomal protection proteins of the ABC-F family. The gene *optrA* was first identified in *E. faecalis* and *E. faecium* and later found in various other gram-positive bacteria [20, 21], while *poxtA* was recently identified on the MRSA (methicillin-resistant *Staphylococcus aureus*) chromosome [22].

*S. lentus* is a coagulase-negative staphylococcus that belongs to the *Staphylococcus sciuri* group (*S. sciuri, S. lentus*, and *S. vitulinus*) [23]. *S. lentus* was traditionally considered to be an animal pathogen and has been isolated from a wide range of pets, farm animals, wild animals, and retail meats [24]. *S. lentus* has also been identified as the causative organism in several serious human infections, including sinusitis**,** endocarditis, peritonitis, septic shock, urinary tract infection, and wound infections, and its clinical significance is apparently increasing [25–27]. In this work, in addition to detecting the florfenicol resistance levels and resistance genes of 39 *Staphylococcus* isolates from poultry and seafood, we also investigated the molecular mechanism of florfenicol resistance of a *S. lentus* strain with high level florfenicol resistance isolated from a hen. Through whole genome sequencing, we found, for the first time, two copies of the genes *cfr* and *fexA* colocalized on a plasmid as well as the chromosome of a bacterium.

## Materials and methods

### Bacteria and antimicrobial susceptibility testing

28 CoNS strains were isolated from fresh fecal samples of domestic animals (ducks, cows, chickens and pigs) collected from several farms in Sichuan, Zhejiang, Shanxi, Shandong and Henan provinces, China, in 2016. 11 CoNS strains were isolated from fresh seafood (including fishes and prawns) intestinal contents from fishfarms in Wenzhou, Zhejiang, China, in 2018. The isolates were identified by Gram’s staining and serum coagulase testing in strict accordance with experimental procedures [28] and verified by homology comparisons of the 16S rRNA genes. Antimicrobial susceptibility was evaluated by the agar dilution method following the guidelines recommended by the Clinical and Laboratory Standards Institute (CLSI, 2017: M100, https://clsi.org/standards/). The MIC of linezolid was determined by the agar dilution method according to the European Committee on Antimicrobial Susceptibility Testing (EUCAST, http://www.eucast.org). *S. aureus* ATCC29213 was used as a control strain.

### Clonal relationship analysis of the strains resistant to florfenicol

The clonal relatedness of the 23 florfenicol-resistant strains (florfenicol MIC ≥ 32 µg/mL) was examined by the pulsed-field gel electrophoresis (PFGE) analysis. *Salmonella enterica* serovar Braenderup H9812 genome was used as a size standard. The bacterial genomic DNA was digested with 40 U of *Sma*I (Takara, Dalian, China). The gel was then electrophoresed in a CHEF-Mapper system (Bio-Rad, USA) and the Bio-Rad Quantity One software (Bio-Rad, USA) was used to analyze the PFGE result. A minimum spanning tree was constructed using a categorical coefficient with the unweighted pair group method with arithmetic mean (UPGMA) clustering.

### Detection of florfenicol resistance genes

The florfenicol resistance genes (*fexA, fexB, cfr, optrA, pexA* and *floR*) were detected by PCR with the primers previously reported (Table 1). Genomic DNA was extracted from each of the 39 isolates using the AxyPrep Bacterial Genomic DNA Miniprep kit (Axygen Scientific, Union City, CA, USA) and used as the template for PCR amplification. Positive amplification products were verified by sequencing with an ABI 3730 automated sequencer (Shanghai Sunny Biotechnology Co., Ltd., Shanghai, China), and the sequencing results were compared with BLAST against the corresponding resistance gene sequences in NCBI nucleotide database (https://blast.ncbi.nlm.nih.gov/blast.cgi).

**Table 1:** Primer sequences and PCR product sizes of the florfenicol resistance genes

Primer	Sequence (5′–3′)	Amplicon size (bp)	Annealing temperature (°C)	References
*floR*1-F	ATGACCACCACACGCCCCGCGTGGGC	1198	58	[7]
*floR*1-R	CTTCGATCCCGCGACGTTCCTTCCGAGA			
*fexA*1-F	CTCTTCTGGACAGGCTGGAA	332	57	[6]
*fexA*1-R	CCAGTTCCTGCTCCAAGGTA			
*fexB*1-F	ACTGGACAGGCAGGCTTAAT	319	57	[8]
*fexB*1-R	CCTGCCCCAAGATACATTGC			
*cfr*1-F	GGGAGGATTTAATAAATAATTTTGGAGAAACAG	580	58	[7]
*cfr*1-R	CTTATATGTTCATCGAGTATATTCATTACCTCATC			
*optrA*1-F	CTTATGGATGGTGTGGCAGC	309	56	[11]
*optrA*1-R	CCATGTGGTTTGTCGGTTCA			
*pexA*1-F	GTTGTGGTCTTTGGCCAGAG	318	56	[9]
*pexA*1-R	TCCATCAAGAGGACACCACC			

### Sequencing and annotation of the *S. lentus* H29 genome

The genomic DNA of *S. lentus* H29 was extracted as mentioned above and sequenced with Illumina HiSeq 2500 and Pacific Bioscience sequencers at Annoroad Gene Technology Co., Ltd. (Beijing, China). The Pacific Bioscience sequencing reads of approximately 10–20 kb in length were assembled by SOAPdenovo v2.04, Celera Assembler 8.0 [29]. Two FASTQ sequence files corresponding to the reads derived from HiSeq 2500 sequencing were used to control assembly quality and to correct possible misidentified bases. Glimmer3.02 software with default parameters was used to predict potential open reading frames (ORFs). ORF annotation was determined by performing BLASTX comparisons with the NCBI nonredundant protein database. Comparisons of nucleotide sequences and amino acid sequences were performed by BLASTN and BLASTP, respectively [30]. BLASTp was applied to compare amino acid sequences with those in the Antibiotic Resistance Genes Database (ARDB, https://card.mcmaster.ca/). The map of the plasmid with GC content and GC skew was drawn with the online CGView Server (http://stothard.afns.ualberta.ca/cgview_server/) and local GView 1.7 with a visual interface [31]. The plasmid sequences used in this study were downloaded from the NCBI database (http://www.ncbi.nlm.nih.gov). The rRNA gene sequences were annotated by the online tool RNAmmer (http://www.cbs.dtu.dk/services/RNAmmer/) [32], and the tRNA sequences were annotated by the online tool tRNAscan-SE 2.0 (http://lowelab.ucsc.edu/tRNAscan-SE/) [33]. Promoter sites were predicted by using Soft Berry BPROM software (http://linux1.softberry.com/berry.phtmltopic=bprom&group=programs&subgroup=gfindb).

### Comparative genomics analysis

Sequences containing resistance genes were obtained from the NCBI nucleotide database by the BLASTN program using the resistance gene sequences of *S. lentus* H29 as the query. The resulting sequences were filtered, and only sequences containing complete resistance genes were retained. CD-HIT was used to cluster the retained sequences using the genome sequence of *S. lentus* H29 as the reference with an identity of ≥ 90%. The sequence sharing the greatest similarity to the other sequences in each cluster was chosen as the candidate for ortholog analysis. Orthologous groups of the genes from the candidate sequences were identified using BLASTP [30]. Sequence retrieval, statistical analysis and other bioinformatics tools used in this study were applied with Perl and Bioperl scripts (http://www.perl.org/).

## Results

### Bacterial strains and antimicrobial susceptibility testing

A total of 39 CoNS strains including 9 species were analyzed in this work (Additional file 1: Table S1). Among them, 28 strains were isolated from animal feces and 11 strains were isolated from the seafood intestinal contents. The strains included *S. epidermidis* (4), *S. lentus* (2), *S. equorum* (6), *S. saprophyticus* (7), *S. sciuri* (4), *S. haemolyticus* (3), *S. gallinarum* (2), *S. cohnii* (3), *S. warneri* (4) and 4 unclassified ones. The results of the antimicrobial susceptibility testing of the strains to 21 antimicrobial agents showed that the strains isolated from the animals generally showed wider resistance spectra and higher MIC levels than those isolated from seafood. More than 60% (17/28) of the animal strains showed resistance to 6 antibiotics, including chloramphenicol (85.8%, 24/28), florfenicol (82.1%, 23/28), clindamycin (75.0%, 21/28), tetracycline (67.9%, 19/28), streptomycin (64.3%, 18/28) and erythromycin (60.7%, 17/28), while the seafood bacteria were only resistant to erythromycin (63.6%, 7/11) (Table 2, Additional file 2: Table S2). Meanwhile, more than 90% of the animal isolates were sensitive to eight other antibiotics, especially amikacin, trimethopim and tigecycline with all the strains sensitive to them. However, the seafood isolates only showed certain resistance rates to erythromycin (63.6%, 7/11) and clindamycin (36.4%, 4/11), and most strains were totally sensitive to some antibiotics, such as linezolid, cefoxitin, vancomycin and norfloxacin (Table 2).

**Table 2:** Characterization of the sensitivity of 39 CoNS isolates to 21 antibiotics

Antibiotics	Animal (N = 28)			Seafood (N = 11)			Total (N = 39)		
	S	I	R	S	I	R	S	I	R
LZD	24 (85.8%)	2 (7.1%)	2 (7.1%)	11 (100%)	0 (0)	0 (0%)	35 (89.8%)	2 (5.1%)	2 (5.1%)
FD	18 (64.3%)	0 (0)	10 (36.7%)	8 (72.7%)	0 (0)	3 (27.3%)	26 (66.7%)	0 (0)	13(33.3%)
CLI	7 (25.0%)	0 (0)	21(75.0%)	7 (63.6%)	0 (0)	4 (36.4%)	14 (35.9%)	0 (0)	25(64.1%)
AMK	28 (100%)	0 (0)	0 (0)	11 (100%)	0 (0)	0 (0)	39 (100%)	0 (0)	0 (0)
ERY	11 (39.3%)	0 (0)	17 (60.7%)	4 (36.4%)	0 (0)	7 (63.6%)	15 (38.5%)	0 (0)	24(61.5%)
GEN	27 (96.4%)	0 (0)	1 (4.6%)	11 (100%)	0 (0)	0 (0)	38 (97.4%)	0 (0)	1(2.6%)
OXA	24(86.%)	0 (0)	4 (14%)	9 (81.8%)	0 (0)	2 (18.2%)	33 (84.6%)	0 (0)	6(15.4%)
FOX	26 (93%)	0 (0)	2 (7%)	11 (100%)	0 (0)	0 (0)	37 (94.9%)	0 (0)	2(5.1%)
RIF	24 (85.8%)	0 (0)	4 (14.2%)	11 (100%)	0 (0)	0 (0)	35 (89.8%)	0 (0)	4(10.2%)
TMP	28 (100%)	0 (0)	0 (0%)	11 (100%)	0 (0)	0 (0)	39 (100.0%)	0 (0)	0 (0)
TET	9 (32.1%)	0 (0)	19 (67.9%)	9 (81.8%)	0 (0)	2 (18.2%)	18 (46.2%)	0 (0)	21(53.8%)
VAN	27 (96.4%)	0 (0)	1 (3.6%)	11 (100%)	0 (0)	0 (0)	38 (97.4%)	0 (0)	1(2.6%)
CLR	17 (60.7%)	0 (0)	11 (39.3%)	8 (72.7%)	0 (0)	3 (27.2%)	25 (64.1%)	0 (0)	14(35.9%)
CHL	4 (14.2%)	0 (0)	24 (85.8%)	10 (90.9%)	0 (0)	1 (9.1%)	14 (35.9%)	0 (0)	25(64.1%)
LVX	21 (75.0%)	0 (0)	7 (25.0%)	10 (90.9%)	0 (0)	1 (9.1%)	31 (79.5%)	0 (0)	8(20.5%)
NOR	23 (82.1%)	0 (0)	5 (17.9%)	11 (100%)	0 (0)	0 (0)	34 (87.2%)	0 (0)	5(12.8%)
KAN	21 (75.0%)	0 (0)	7 (25.0%)	9 (81.8%)	0 (0)	2 (18.2%)	30 (76.9%)	0 (0)	9(23.1%)
TGC	28 (100%)	0 (0)	0 (0)	11 (100%)	0 (0)	0 (0)	39 (100%)	0 (0)	0 (0)
TEC	27 (96.4%)	0 (0)	1 (4.6%)	11 (100%)	0 (0)	0 (0)	38 (97.4%)	0 (0)	1(2.6%)
STR	10 (35.7%)	0 (0)	18 (64.3%)	10 (90.9%)	0 (0)	1 (9.1%)	20 (51.3%)	0 (0)	19(48.7%)
FFC	5 (17.9%)	0 (0)	23 (82.1%)	11(100%)	0 (0)	0 (0)	16 (41.0%)	0 (0)	23(59.0%)

LZD, Linezolid; FD, Fusidic Acid; OXA, Oxacillin; TGC, Tigecycline; LVX, Levofloxacin; FOX, Cefoxitin; TMP, Trimethopim; CHL, Chloramphenicol; TEC, teicoplanin; FFC, Florfenicol; CLR, Clarithromycin; CLI, Clindamycin; RIF, Rifampin; NOR, Norfloxacin; VAN, Vancomycin; GEN, Gentamycin; TET, Tetracycline; STR, Streptomycin; AMK, Amikacin; KAN, Kanamycin; ERY, Erythromycin

### Identification of florfenicol resistance genes in the CoNS isolates

In this work, of all 6 florfenicol resistance-related genes (*fexA*, *cfr, optrA, floR, fexB* and *pexA*), only 3 (*fexA, cfr* and *fexB*) were identified in the 39 *Staphylococcus* strains. A total of 37 genes, including 26 *fexA, 6 cfr* and 5 *fexB* genes, were identified in 27 strains, with one (*S. cohnii* H19) and 16 strains each carrying a *cfr* and a *fexA* genes, respectively, 5 strains carrying both a *fexA* and a *cfr* genes, and other 5 isolates harboring both a *fexA* and a *fexB* genes*,* while the remaining twelve strains were free of the resistance gene. Strains from animals presented a much higher positive rate and carried much more resistance genes, with 82.1% (23/28) of the strains carrying 91.9% (34/37) of the resistance genes, while in the seafood bacteria, only three strains (3/11, 27.3%) carried one *fexA* gene each (3/37, 8.1%). All 23 florfenicol-resistant isolates (florfenicol MIC level ≥ 32 µg/mL) were isolated from animals, and they all carried two (*fexA* and *fexB*) or one (*fexA*) florfenicol resistance gene. Among the 16 florfenicol-sensitive isolates (MIC ≤ 1 µg/mL), 12 were free of the florfenicol resistance gene, and 3 (HXM5, HXM10 and HXM13 all isolated from seafood) carried a *fexA* gene and one strain from poultry with a *cfr* gene. Among the 5 isolates that carried both *fexA* and *cfr*, two strains (*S. sciuri* FC11 and *S. haemolyticus* FC24) showed an MIC value of 8 μg/mL to linezolid, which was interpreted as an intermediate for linezolid, while the other three strains showed MIC values of ≤ 0.25 μg/mL for linezolid.

### Clonal relatedness of the florfenicol-resistant CoNS isolates

**Fig. 1:**
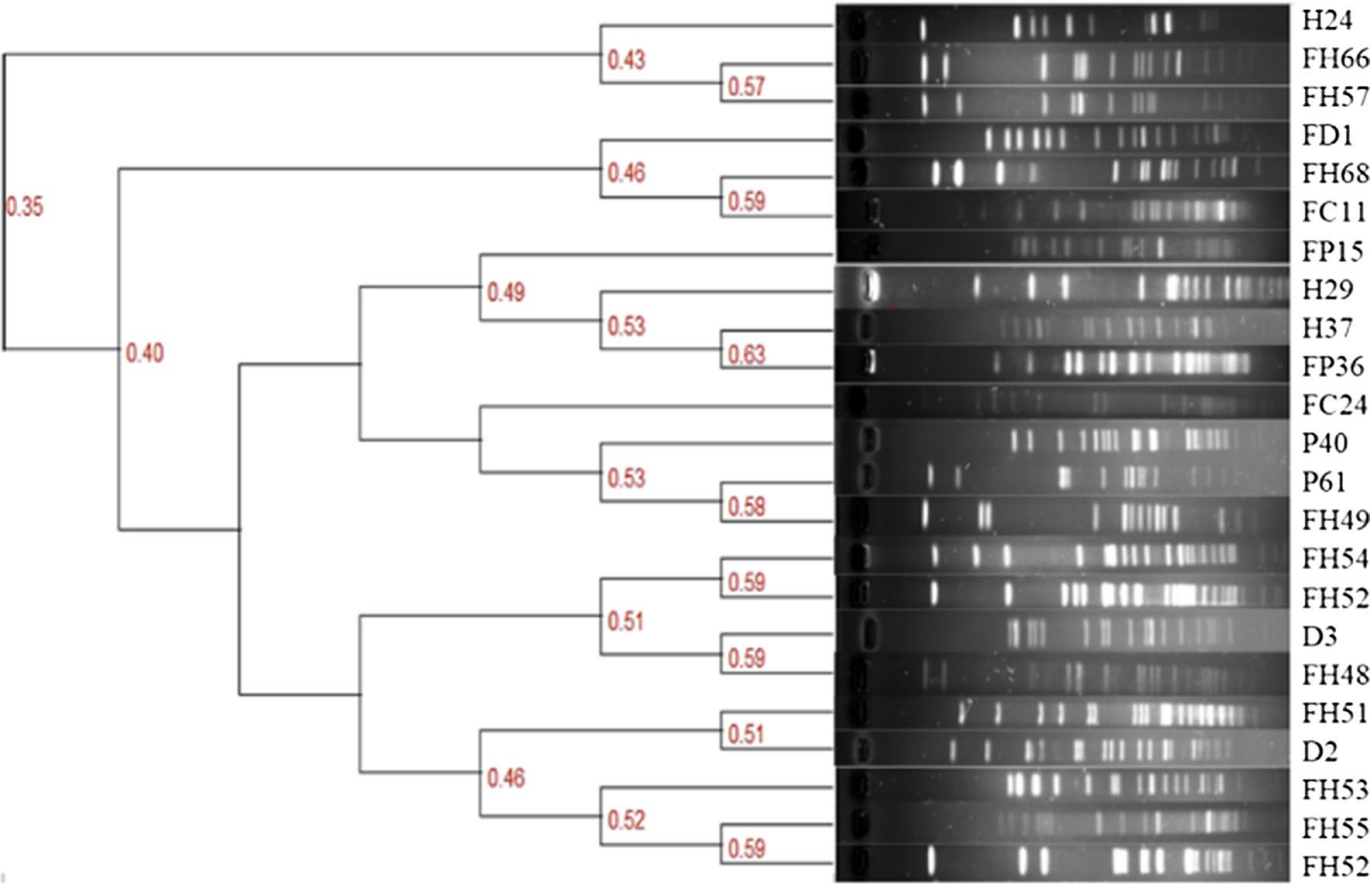
PFGE patterns of 23 florfenicol-resistant CoNS isolates

Clonal relationship analysis for 23 florfenicol-resistant strains (MIC ≥ 32 µg/mL) revealed that no clonal relatedness was identified among them, including the strains of the same species (Fig. 1). The highest similarity of 63% was observed between two strains of different species, *S. equorum* (H37) and *S. haemolyticus* (FP36), which were isolated from different hosts (hen and pig, respectively).

### General features of the *S. lentus* H29 genome

To analyze the molecular characteristics of the florfenicol-resistant CoNS strains, *S. lentus* H29, co-carrying *fexA* and *cfr* with a wide resistance spectrum and high MIC values to the antibiotics tested, was chosen for whole genome sequencing (WGS) analysis. The general features of the H29 genome are shown in Table 3. The complete genome of *S. lentus* H29 consists of one chromosome and two plasmids (pH29-46 and pH29-26). The chromosome with a G + C content of 31.9% was 2,802,282 bp in length encoding 2683 ORFs. pH29-46 was 46,167 bp in length and encoded 46 ORFs, and pH29-26 was 26,210 bp in length encoding 26 ORFs. The whole genome of *S. lentus* H29 encoded 11 resistance genes, of which 6 [*cfr, fexA, ant(6)-Ia*, *aacA-aphD, mecA* and *mph(C)*] were encoded on the chromosome, 4 [*cfr, fexA, aacA-aphD* and Δ*tcaA*] on pH29-46 and 1 (*fosD*) on pH29-26. The resistance phenotypes coincided with the resistance genotypes (Table 4). In addition to showing resistance to florfenicol (MIC of 256 μg/mL) and chloramphenicol (MIC of 256 μg/mL), *S. lentus* H29 was also resistant to erythromycin (> 64 μg/mL) and macrolide antibiotics.

**Table 3:** General characteristics of the *S. lentus* H29 genome

	Chromosome	pH29-46	pH29-26
Size (bp)	2,802,282	46,167	26,210
GC content (%)	31.90	29.73	31.94
Predicted CDs	2741	46	30
Known proteins	1929	33	20
Hypothetical proteins	812	13	10
Protein coding sequences (%)	87.30	82.33	87.54
Average ORF length (bp)	892	719	878

**Table 4:** Antimicrobial resistance determinants in *S.lentus* H29

Antibiotics class	Antibiotics tested	MIC (µg/mL)	Interpretation	Resistance genes
Macrolide	Erythromycin	> 64	R	*erm(ABC)*
Lincosamide	Clindamycin	> 64	R	
	Clarithromycin	> 64	R	
	Streptomycin	64	R	
Aminoglycosides	Gentamycin	4	S	*aac-aph*, *ant-Ia*
	Amikacin	4	S	
	Kanamycin	> 64	R	
β-lactam	Cefoxitin	2	R	*mecA*, *mecC*
	Oxacillin	2	R	
Fusidic Acid	Fusidic Acid	1	S	
Rifampicin	Rifampin	> 64	R	*rpoB*
FLuoroquinolones	Norfloxacin	> 64	R	*norA*
	Levofloxacin	4	R	*gyrA*, *gyrB*
Phenicol	Chloramphenicol	256	R	*cml*
	Florfenicol	256	R	*cfr*, *fexA*
Sulfonamides/Trimethoprim	Sulfonamides/Trimethoprim	1	S	
Tetracycline	Tetracycline	64	R	*tet(K),* *tet(L)*
	Tigecycline	2	S	
Oxazolidinones	Linezolid	< 0.125	S	
Glycopeptides	Vancomycin	2	S	
	Teicoplanin	0.5	S	

### Comparative genomics analysis of the resistance plasmids and the *fexA-* and *cfr*-related sequences in the *S. lentus* H29 genome

**Fig. 2:**
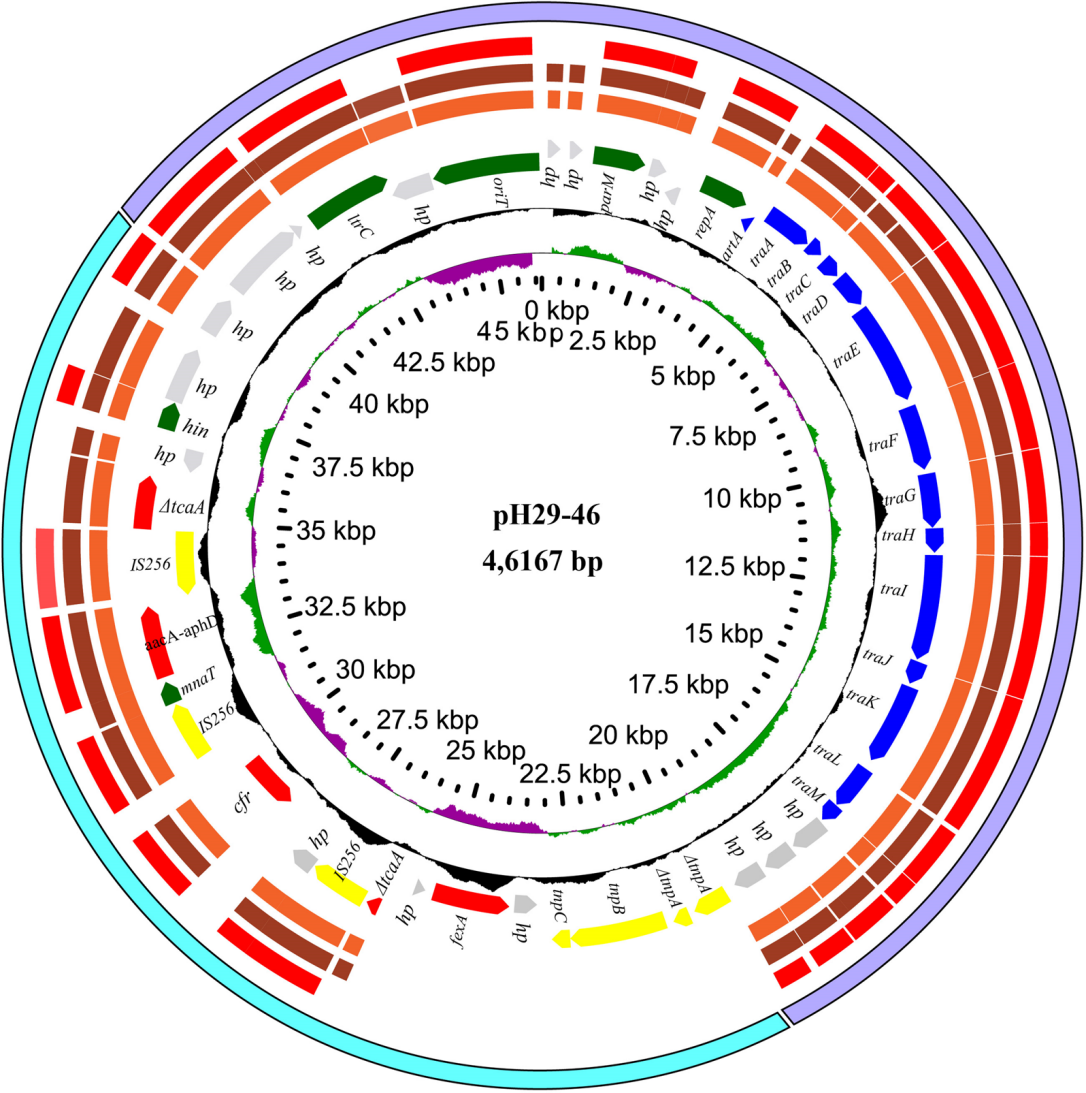
Genetic map of pH29-46 and its comparison with other plasmids of the highest nucleotide sequence similarities. From the outside to the inside: circle 1, pH29-46 region A in purple and region B in green; circle 2, pSX01 (the plasmid of *S. xylosus* strain 378 isolated from pig, NZ_KP890694.1); circle 3, pSR01 (the plasmid of *S. aureus* strain SR434 isolated from human, NZ_CP019564.1); circle 3, pLRSA417 (*S. aureus* strain 417 isolated from human, KJ922127.1); circle 4, pH29-46 with genes encoded on the two strands. The red arrows indicate drug-resistant genes, blue arrows indicate transfer genes and the gray arrows indicate the genes encoding hypothetical proteins

Three plasmids, pSX01 (NZ_KP890694.1) of *Staphylococcus xylosus* 378, pSR01 (NZ_CP019564.1) of *S-taphylococcus aureus* strain SR434 and pLRSA417 (KJ922127.1) of *Staphylococcus aureus* 417, sharing the highest nucleotide sequence similarities (coverage > 70%, identities ≥ 97%) with pH29-46 were retrieved from the NCBI nucleotide database. According to the structure and function of the genes encoded on the plasmid, pH29-46 could be divided into two regions (Regions A and B, Fig. 2). Region A was about 26 kb in size encoding the backbone genes, mainly including a replication gene *repA*, a segregation gene *parM*, 16 T4SS genes and several hypothetical protein genes, and it displayed 98–100% identity to the corresponding regions of the plasmids pSR01 and pLRSA417. Region B, about 20 kb in length, harbored five resistance genes, which could be divided into two segments. One segment (about 7.5 kb in length) included the *tnpABC* and *fexA* genes, which were not present in the three plasmids from the database. The other segment was a 12.5 kb sequence encoding the resistance genes of *cfr*, *aacA-aphD* and *tcaA*, and three copies of IS*256* showing 99% identity and 80% coverage to the sequence on pSR01 and pLRSA417.

**Fig. 3:**
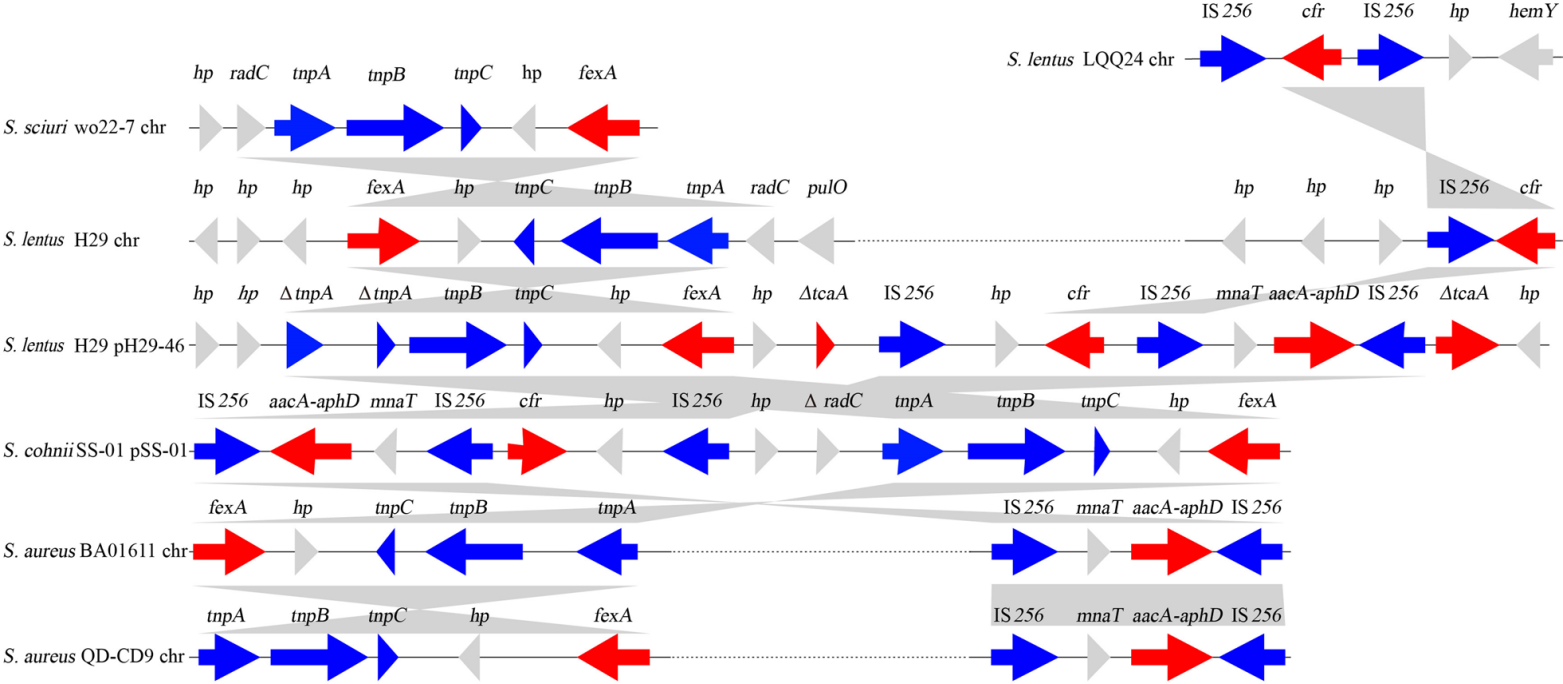
Genetic environments of the *fexA* and *cfr* genes encoded in plasmids or S. LQQ24chromosomes. The sequences and their origins are: *S. lentus* S. LQQ24 chr (the chromosome of *S. lentus* S. LQQ24 isolated from chicken in China, KF029594.1), *S. sciuri* wo227 chr (the chromosome of *S. sciuri* wo227 isolated from swine, KX982170.1), *S. lentus* H29 chr (the chromosome of H29 isolated from hen of this work, CP059679), *S. lentus* H29 pH29-46 (the plasmid of pH29-46 isolated from a hen of this work, CP059680), *S. cohnii* pSS-01 (the plasmid of S. cohnii SS-01 isolated from swine, JQ041372.1), *S.aureus* BA01611 chr (the chromosome of *S.aureus* BA01611 isolated from bovine, CP019945.1), *S.aureus* QD-CD9 chr (the chromosome of *S.aureus* QD-CD9 isolated from in swine, CP031838.1). Antimicrobial resistance genes are in red, transposase or integrase genes are in blue and other genes are in gray. Gray-shaded areas represent regions with > 95% nucleotide sequence identities. The arrows indicate the positions and orientations of the genes

It turned out that the *S. lentus* H29 genome carried two identical copies of the gene arrays of *radC*-*tnpABC*-hp-*fexA* (5671 bp) and IS256-*cfr* (2690 bp), of which one copy was encoded on plasmid pH29-46, while the other was encoded on the chromosome (Fig. 3). To the best of our knowledge, this is the first case that the combination of the mobile genetic element related *cfr* (IS*256*-*cfr)* and *fexA* (*tnpABC-hp-fexA*) was identified in both the plasmid (pH29-46) and the chromosome of an isolate *S. lentus* H29, respectively, even though this combination has been identified in several other plasmids such as pSS-01 of *S. cohnii* (JQ041372.1) and either IS*256*-*cfr* or *tnpABC-hp-fexA* has been identified encoded in plasmids or chromosomes in other *Staphylococcus* strains of different source (Fig. 3).

## Discussion

In this work, of the 39 CoNS strains from 9 species analyzed, the *S. saprophyticus* strains, with the most isolates (17.95%, 7/39), were isolated from both the animals and seafood, which was in accordance with the statistics reported [34]. *S. epidermis* that has been reported to be most commonly isolated from humans [35], was present in the animals as well as seafood. It was found that the isolates from the animals demonstrated wider resistance spectra and higher MIC levels than those isolated from seafood. Although most antibiotic resistance rates of the animal CoNS isolates were similar to those previously reported, the resistance rates for clarithromycin (39.3%, 11/28) and fusidic acid (36.7%, 10/28) were higher than those in recent publications [36], which may indicate the abused use of the drugs in local livestock husbandry.

Of the 39 isolates, 69.2% (27/39) carried one or two florfenicol resistance-related genes, with 26 carrying a *fexA* gene*,* 6 carrying a *cfr* gene and 5 with *a fexB* gene, respectively. Many studies have reported that *fexA* is one of the most common florfenicol resistance gene in household animals in rural China [6, 11, 37]. In this study, the *fexA* gene occupied 70.3% (26/37) of the florfenicol resistance genes. The isolates from animals carried much more resistance genes (91.9%, 34/37) than those from the seafood (3/11, 27.3%), and all 23 florfenicol-resistant isolates were from the animals. It was interesting to find that of the 5 isolates each with both *fexA* and *cfr*, two strains presented an intermediate resistance for linezolid (with MIC levels of 8 μg/mL), much higher than those of the other three (with the MIC values of ≤ 0.25 μg/mL). According to previous reports, linezolid resistance was related with ATP-binding cassette transporter gene *optrA* and it has been identified in bacteria of the animal origin [38, 39]. However, in this work, the *optrA* gene has not been identified in these strains. This may indicate that other mechanisms rather than *optrA* conferring the low-level linezolid resistance might exist in the two isolates.

At present, except for *S. lentus* H29 of this work, no complete genome sequence of *S. lentus* is available in the NCBI nucleotide database. The whole genome of *S. lentus* H29 encoded 11 resistance genes, including two copies of the mobile genetic elements (MGEs) related florfenicol resistance genes *cfr* (IS256-*cfr*) and *fexA* (*radC*-*tnpABC*-hp-*fexA*) with one copy of them encoded in the chromosome and the other in the plasmid*.* This is the first case of one strain carrying two identical copies of *cfr* and *fexA* related MGEs, even though these MGEs could be found encoded in either the chromosome or the plasmid of the different bacterial species [40, 41]. It indicated that the MGEs carried florfenicol resistance genes could be horizontally transferred between strains of different species, causing the spread of drug resistance. On the other hand, these MGE-related florfenicol resistance genes identified in bacteria of different origins (such as those isolated from animals and humans) may demonstrate the threat of the use of antibiotics in animals to human health.

## Conclusions

In this work, the animal CoNS isolates showed wider resistance spectra and higher resistance levels to multiple antibiotics than those of seafood-derived isolates. The main molecular mechanism that makes the CoNS isolates resistant to florfenicol is the *fexA, fexB* and *cfr* genes. Sequncing analysis of the *S. lentus* H29 genome showed that the *fexA* and *cfr* genes were related with the mobile genetic elements and located on both the plasmid and the chromosome which indicated that they may transmit between different bacterial species and cause widespread of florfenicol resistance.

## Supplementary Information

**Additional file 1: Table S1.** Resistance phenotype and florfenicol resistance genes of the CoNS isolates.

**Additional file 1: Table S2.** Antibiotics resistance profile of all 39 CoNS isolates.

## Data Availability

All data generated or analyzed during this study are included in this published article and its supplementary information files. The data related to the paper are deposited in the NCBI GenBank. The accession numbers for the chromosome, pH29-46 and pH29-26 are CP059679, CP059680and CP059681, respectively.

## Abbreviations

CoNS: Coagulase-negative *Staphylococcus*
BLAST: The basic local alignment search tool
MIC: Minimum inhibitory concentration
PFGE: Pulsed-field gel electrophoresis
PCR: Polymerase chain reaction
UPGMA: Unweighted pair-group method with arithmetic means
