# Supplementary material for: Characterization of florfenicol resistance genes in the coagulase-negative Staphylococcus (CoNS) isolates and genomic features of a multidrug-resistant Staphylococcus lentus strain H29
Source: Antimicrob Resist Infect Control. 2021 Jan 7;10:9. doi: 10.1186/s13756-020-00869-5 (PMC7791814; doi:10.1186/s13756-020-00869-5)
Supplement: Supplementary file 1 — Additional file 1: Table S1. Resistance phenotype and florfenicol resistance genes of the CoNS isolates. [file 13756_2020_869_MOESM1_ESM.docx]

Table S1. Resistance phenotype and florfenicol resistance genes of the CoNS isolates

| Strains | Species | Resistance phenotype | Resistance genes |
| --- | --- | --- | --- |
| HXM5 | *S. epidermidis* | None | *fexA* |
| HXM6 | *S. warneri* | ERY | none |
| HXM7 | *S. warneri* | CLI, ERY | none |
| HXM10 | *S. warneri* | CLR, ERY, KAN | *fexA* |
| HXM13 | *uncategorized* | None | *fexA* |
| HXM15 | *S. saprophyticus* | ERY, STR | none |
| HXM16 | *S. sciuri* | ERY | none |
| HXM25 | *uncategorized* | CLR, ERY | none |
| HXM63 | *S. warneri* | CLR, ERY | none |
| HXM64 | *S. epidermidis* | CM, KAN | none |
| HXM68 | *S. saprophyticus* | TET, STR | none |
| FP15 | *S. gallinarum* | CM, FFC, TET, STR | *fexA* |
| FP36 | *S. haemolyticus* | FUS, CLR, CLI, ERY, CM, FFC, TET, STR | *fexA* |
| FC11 | *S. sciuri* | LNZ, FUS, LVX, CLR, CLI, ERY, CM, FFC, RIF, NOR, TET, STR, KAN | *fexA, cfr* |
| FC24 | *S. haemolyticus* | LNZ, CLI, ERY, TEC, CM, FFC, VAN, STR | *fexA, cfr* |
| FH48 | *S. saprophyticus* | FOX, CM, FFC, TET | *fexA* |
| FH49 | *S. saprophyticus* | ERY, CM, FFC, STR | *fexA, fexB* |
| FH50 | *S. gallinarum* | ERY, CM, FFC, TET, STR | *fexA* |
| FH51 | *S. saprophyticus* | CLR, CLI, ERY, CM, FFC, STR | *fexA* |
| FH52 | *S. saprophyticus* | FUS, FOX, CM, FFC, TET, STR | *fexA, fexB* |
| FH53 | *S. equorum* | ERY, CM, FFC, STR | *fexA, fexB* |
| FH54 | *S. haemolyticus* | CM, FFC | *fexA* |
| FH55 | *S. equorum* | CLI, ERY, CM, FFC, TET | *fexA* |
| FH57 | *uncategorized* | OXA, LVX, CLR, CLI, ERY, CM, FFC, NOR, TET | *fexA, fexB* |
| FH66 | *S. lentus* | CM, FFC | *fexA* |
| FH68 | *S. equorum* | ERY, CM, FFC, NOR, TET, STR | *fexA* |
| D2 | *S. equorum* | TGC, LVX, CLR, CLI, ERY, CM, FFC, NOR, TET, STR, KAN | *fexA,fexB* |
| D3 | *S. cohnii subsp. cohnii* | FUS, TGC, LVX, FOX, CLR, CLI, CM, FFC, NOR, TET, STR | *fexA* |
| FD1 | *S. sciuri* | FUS, CLR, CLI, ERY, CM, FFC, KAN | *fexA, cfr* |
| H4 | *S. sciuri* | FUS, CM, STR | none |
| H6 | *S. saprophyticus* | FUS | none |
| H19 | *S. cohnii subsp. cohnii* | TGC, OXA, CLI, TET | none |
| H24 | *S. equorum* | CM, FFC | *fexA, cfr* |
| H29 | *S. lentus* | TGC, LVX, CLR, CLI, ERY, CM, FFC, RIF, NOR, TET, STR, KAN | *fexA, cfr* |
| H33 | *S. epidermidis* | TGC, LVX, ERY, TET, STR, KAN | none |
| H37 | *S. equorum* | FUS, CLR, CLI, ERY, CM, FFC, RIF, NOR, STR, KAN | *fexA* |
| C10 | *S. cohnii subsp. cohnii* | None | none |
| P40 | *S. epidermidis* | TGC, LVX, CLR, CLI, ERY, TEC, CM, FFC, NOR, TET, STR, KAN | *fexA, cfr* |
| P61 | *uncategorized* | CLR, CLI, ERY, CM, FFC | *fexA* |

LZD, Linezolid; FD, Fusidic Acid; OXA, Oxacillin; TGC, Tigecycline; LVX, Levofloxacin; FOX, Cefoxitin; TMP, Trimethopim; CHL,Chloramphenicol; TEC, teicoplanin; FFC, Florfenicol; CLR, Clarithromycin; CLI, Clindamycin; RIF, Rifam; NOR, Norfloxacin; VAN, Vancomycin; GEN, Gentamycin; TET, Tetracycline; STR, Streptomycin; AMK, Amikacin; KAN, Kanamycin; ERY, Erythromycin.
