# Supplementary material for: Characterization of florfenicol resistance genes in the coagulase-negative Staphylococcus (CoNS) isolates and genomic features of a multidrug-resistant Staphylococcus lentus strain H29
Source: Antimicrob Resist Infect Control. 2021 Jan 7;10:9. doi: 10.1186/s13756-020-00869-5 (PMC7791814; doi:10.1186/s13756-020-00869-5)
Supplement: Supplementary file 2 — Additional file 1: Table S2. Antibiotics resistance profile of all 39 CoNS isolates. [file 13756_2020_869_MOESM2_ESM.docx]

Table S2. Antibiotics resistance profile of all 39 CoNS isolates

| Strain | LLD | FD | OXA | TGC | LVX | FOX | CLR | CLD | ERY | TEC | TMP | CHL | FFC | RIF | NOR | VAN | GEN | TET | STR | AMK | KAN |
| --- | --- | --- | --- | --- | --- | --- | --- | --- | --- | --- | --- | --- | --- | --- | --- | --- | --- | --- | --- | --- | --- |
| ATCC29213 | ＜0.125 | 2 | ＜0.125 | ＜0.125 | ＜0.125 | 2 | ＜0.125 | 0.25 | 0.25 | ＜0.125 | ＜0.125 | 8 | 1 | ＜0.125 | 0.5 | 2 | ＜0.125 | ＜0.125 | 1 | 1 | 0.5 |
| HXM5 | 0.25 | 0.25 | ＜0.125 | 0.25 | 0.25 | 1 | ＜0.125 | 0.25 | 0.5 | 2 | 4 | 8 | 1 | ＜0.125 | 0.5 | 4 | ＜0.125 | 0.25 | 2 | 1 | 0.5 |
| HXM6 | 0.25 | 0.25 | ＜0.125 | 0.25 | 0.25 | 2 | 4 | 0.25 | >64 | 0.25 | 4 | 8 | 1 | ＜0.125 | 1 | 4 | ＜0.125 | 0.25 | 2 | 1 | 0.5 |
| HXM7 | 2 | 1 | 0.5 | 0.25 | 2 | 2 | ＜0.125 | 16 | 64 | 0.5 | 8 | 8 | 1 | ＜0.125 | 2 | 2 | ＜0.125 | 0.25 | 2 | 1 | 0.5 |
| HXM10 | 2 | 0.25 | 4 | 0.25 | 2 | 4 | 32 | ＜0.125 | >64 | 0.5 | 8 | 8 | 1 | ＜0.125 | 2 | 2 | ＜0.125 | 0.25 | 2 | 1 | >64 |
| HXM13 | 0.25 | 4 | ＜0.125 | ＜0.125 | 2 | 2 | ＜0.125 | 8 | 2 | 0.5 | ＜0.125 | 8 | 1 | ＜0.125 | 4 | 2 | ＜0.125 | 0.25 | 2 | 0.5 | 0.25 |
| HXM15 | ＜0.125 | 1 | ＜0.125 | ＜0.125 | ＜0.125 | 0.5 | ＜0.125 | 0.25 | >64 | ＜0.125 | ＜0.125 | 4 | 1 | ＜0.125 | 8 | 2 | ＜0.125 | ＜0.125 | 64 | 1 | 0.25 |
| HXM16 | 0.25 | 4 | ＜0.125 | ＜0.125 | 2 | 2 | ＜0.125 | 8 | >64 | 0.5 | 4 | 8 | 1 | ＜0.125 | 4 | 2 | ＜0.125 | 0.25 | 4 | 1 | 0.25 |
| HXM25 | 2 | 0.5 | ＜0.125 | 0.5 | 4 | 2 | 16 | ＜0.125 | >64 | 1 | 8 | 8 | 1 | ＜0.125 | 2 | 4 | ＜0.125 | 16 | 2 | 1 | 0.25 |
| HXM63 | 0.25 | 0.25 | ＜0.125 | 0.25 | 2 | 2 | 32 | 8 | >64 | 1 | 1 | 8 | 1 | ＜0.125 | 1 | 4 | ＜0.125 | 0.25 | 2 | 1 | 0.5 |
| HXM64 | ＜0.125 | 1 | ＜0.125 | ＜0.125 | 2 | 2 | ＜0.125 | ＜0.125 | 0.5 | 1 | 4 | 32 | 1 | 0.5 | 1 | 4 | ＜0.125 | 0.25 | 2 | 1 | >64 |
| HXM68 | ＜0.125 | 4 | ＜0.125 | ＜0.125 | ＜0.125 | 2 | ＜0.125 | 0.25 | 2 | ＜0.125 | ＜0.125 | 1 | 1 | ＜0.125 | 1 | 4 | 0.5 | 64 | 64 | 0.5 | 0.25 |
| FP15 | ＜0.125 | 4 | ＜0.125 | ＜0.125 | ＜0.125 | 2 | ＜0.125 | 8 | 2 | ＜0.125 | 1 | 256 | 64 | ＜0.125 | 1 | 4 | ＜0.125 | >64 | >64 | 0.5 | 8 |
| FP36 | 0.25 | 4 | ＜0.125 | 0.5 | 2 | 2 | >64 | >64 | >64 | 0.25 | 8 | 64 | 64 | ＜0.125 | 2 | 2 | ＜0.125 | 16 | 32 | 1 | 8 |
| FC11 | 8 | 4 | 0.5 | 0.5 | >16 | 2 | >64 | >64 | >64 | 0.25 | 8 | 256 | 256 | >64 | >64 | 2 | 16 | 64 | 64 | 2 | >64 |
| FC24 | 8 | ＜0.125 | ＜0.125 | 0.5 | ＜0.125 | 1 | 0.25 | >64 | >64 | 64 | ＜0.125 | 128 | 64 | ＜0.125 | ＜0.125 | >64 | 0.25 | 0.25 | 64 | 1 | 0.5 |
| FH48 | ＜0.125 | 0.5 | ＜0.125 | ＜0.125 | ＜0.125 | 16 | ＜0.125 | 8 | 0.5 | ＜0.125 | ＜0.125 | 128 | 64 | ＜0.125 | 0.25 | 2 | ＜0.125 | 16 | 2 | 0.5 | 0.25 |
| FH49 | ＜0.125 | 0.5 | ＜0.125 | ＜0.125 | ＜0.125 | 2 | ＜0.125 | 8 | >64 | ＜0.125 | ＜0.125 | 256 | 64 | ＜0.125 | ＜0.125 | 2 | 0.5 | ＜0.125 | 32 | 0.5 | 0.25 |
| FH50 | ＜0.125 | 0.5 | ＜0.125 | ＜0.125 | ＜0.125 | 1 | ＜0.125 | 8 | >64 | ＜0.125 | ＜0.125 | 256 | 64 | ＜0.125 | 2 | 2 | 0.5 | 16 | 64 | 0.5 | 8 |
| FH51 | 0.25 | 0.5 | ＜0.125 | 0.5 | 2 | 2 | >64 | >64 | >64 | 0.25 | ＜0.125 | 256 | 64 | ＜0.125 | 2 | 2 | 0.5 | 16 | 32 | 0.5 | 8 |
| FH52 | 0.25 | 4 | ＜0.125 | ＜0.125 | ＜0.125 | 4 | ＜0.125 | 8 | 0.5 | ＜0.125 | 4 | 256 | 64 | ＜0.125 | 2 | 2 | 0.5 | 16 | 32 | ＜0.125 | 8 |
| FH53 | ＜0.125 | ＜0.125 | ＜0.125 | ＜0.125 | ＜0.125 | 0.5 | ＜0.125 | 8 | >64 | ＜0.125 | ＜0.125 | 256 | 64 | ＜0.125 | 2 | 2 | ＜0.125 | ＜0.125 | 32 | ＜0.125 | 8 |
| FH54 | ＜0.125 | ＜0.125 | ＜0.125 | ＜0.125 | ＜0.125 | 0.25 | ＜0.125 | 0.5 | 0.25 | ＜0.125 | ＜0.125 | 64 | 64 | ＜0.125 | ＜0.125 | 2 | ＜0.125 | ＜0.125 | ＜0.125 | ＜0.125 | ＜0.125 |
| FH55 | ＜0.125 | ＜0.125 | ＜0.125 | 0.5 | ＜0.125 | 0.25 | 0.25 | >64 | >64 | ＜0.125 | ＜0.125 | 256 | 64 | 0.5 | 1 | 2 | ＜0.125 | >64 | 2 | 0.5 | 8 |
| FH57 | 0.25 | ＜0.125 | 2 | 0.5 | 16 | 2 | >64 | >64 | >64 | ＜0.125 | ＜0.125 | 128 | 128 | ＜0.125 | >64 | 0.5 | 0.5 | >64 | 2 | 0.5 | 8 |
| FH66 | ＜0.125 | 0.5 | ＜0.125 | 0.5 | ＜0.125 | 0.25 | ＜0.125 | 0.25 | 0.25 | ＜0.125 | ＜0.125 | 128 | 64 | ＜0.125 | ＜0.125 | 2 | ＜0.125 | 0.25 | 2 | 1 | 8 |
| FH68 | ＜0.125 | 1 | ＜0.125 | ＜0.125 | ＜0.125 | 0.5 | ＜0.125 | ＜0.125 | >64 | ＜0.125 | ＜0.125 | 64 | 32 | ＜0.125 | >64 | 2 | 0.5 | 16 | 32 | 0.5 | 8 |
| H4 | ＜0.125 | 4 | ＜0.125 | ＜0.125 | ＜0.125 | 1 | >64 | >64 | >64 | ＜0.125 | ＜0.125 | 128 | 64 | >64 | 2 | 2 | ＜0.125 | 16 | 32 | 1 | 64 |
| H6 | ＜0.125 | 4 | ＜0.125 | ＜0.125 | ＜0.125 | 4 | ＜0.125 | 8 | 0.25 | ＜0.125 | ＜0.125 | 32 | 1 | ＜0.125 | 2 | 2 | ＜0.125 | 16 | 64 | 1 | 0.25 |
| H19 | 0.25 | 4 | ＜0.125 | ＜0.125 | ＜0.125 | 2 | ＜0.125 | ＜0.125 | 0.25 | ＜0.125 | ＜0.125 | 1 | 1 | ＜0.125 | 1 | 2 | ＜0.125 | ＜0.125 | 2 | ＜0.125 | ＜0.125 |
| H24 | 0.25 | 4 | 2 | 0.25 | ＜0.125 | 2 | 0.25 | 64 | 2 | ＜0.125 | 1 | 4 | 1 | ＜0.125 | 2 | 2 | ＜0.125 | 64 | 2 | 0.5 | 0.25 |
| H29 | ＜0.125 | ＜0.125 | ＜0.125 | ＜0.125 | ＜0.125 | 0.5 | ＜0.125 | 0.25 | 0.25 | ＜0.125 | ＜0.125 | 64 | 64 | ＜0.125 | ＜0.125 | 4 | ＜0.125 | 2 | 0.25 | 0.5 | 0.25 |
| H33 | ＜0.125 | 1 | ＜0.125 | 2 | 4 | 2 | >64 | >64 | >64 | 0.5 | 1 | 256 | 256 | >64 | >64 | 2 | 4 | 64 | 64 | 4 | >64 |
| H37 | ＜0.125 | 0.25 | ＜0.125 | 2 | 4 | 2 | 4 | 0.25 | >64 | 0.5 | 1 | 8 | 1 | ＜0.125 | 1 | 4 | 4 | 32 | 64 | 4 | >64 |
| C10 | 0.25 | 4 | ＜0.125 | 0.25 | ＜0.125 | 2 | >64 | >64 | >64 | ＜0.125 | ＜0.125 | 128 | 128 | >64 | >64 | 2 | 1 | 32 | 64 | 1 | 64 |
| P61 | 0.25 | 2 | ＜0.125 | 0.25 | 2 | 0.25 | ＜0.125 | 0.25 | 2 | 0.5 | ＜0.125 | 4 | 1 | ＜0.125 | 1 | 2 | ＜0.125 | ＜0.125 | 2 | 1 | 4 |
| P40 | 0.25 | 0.5 | ＜0.125 | 0.25 | 2 | 0.5 | >64 | >64 | >64 | ＜0.125 | ＜0.125 | 64 | 64 | ＜0.125 | 0.25 | 2 | ＜0.125 | ＜0.125 | 2 | ＜0.125 | ＜0.125 |
| D2 | 0.25 | 0.25 | 0.25 | 2 | 4 | 2 | >64 | >64 | >64 | 4 | 8 | 64 | 64 | ＜0.125 | 32 | 4 | 8 | 64 | >64 | 4 | 64 |
| D3 | 0.25 | 0.25 | ＜0.125 | 2 | 4 | 2 | >64 | >64 | >64 | 1 | 1 | 64 | 64 | ＜0.125 | 32 | 4 | 8 | 64 | >64 | 4 | 64 |
| FD1 | 0.25 | 4 | ＜0.125 | 2 | 4 | 8 | >64 | >64 | 0.25 | 1 | 1 | 64 | 64 | ＜0.125 | 32 | 2 | ＜0.125 | 64 | 64 | ＜0.125 | 16 |

LZD, Linezolid; FD, Fusidic Acid; OXA, Oxacillin; TGC, Tigecycline; LVX, Levofloxacin; FOX, Cefoxitin; CLR, Clarithromycin; CLI, Clindamycin; ERY, Erythromycin; TMP, Trimethopim; CHL, Chloramphenicol; TEC, teicoplanin; FFC, Florfenicol; RIF, Rifampin; NOR, Norfloxacin; VAN, Vancomycin; GEN, Gentamycin; TET, Tetracycline; STR, Streptomycin; AMK, Amikacin; KAN, Kanamycin.
